# Cellular Senescence-Associated Gene Expression in Circulating CD4^+^, CD8^+^, CD19^+^ Lymphocytes of HNSCC Patients: Associations with Clinical Parameters

**DOI:** 10.3390/cells15161477

**Published:** 2026-08-18

**Authors:** Kamila Ostrowska, Patryk Niewinski, Igor Piotrowski, Agata Kubicka, Julia Ostapowicz, Julia Kozikowska, Aleksandra Jazikowska, Karolina Czochór, Joanna Marchlewska, Danuta Procyk, Ewa Leporowska, Wiktoria M. Suchorska, Matthew J. Yousefzadeh, Michal M. Masternak, Wojciech Golusiński

**Affiliations:** 1 Department of Clinical Chemistry and Molecular Diagnostics, Poznan University of Medical Sciences, Rokietnicka 8, 60-806 Poznan, Poland; juliakozikowska@icloud.com; 2 Radiobiology Laboratory, Greater Poland Cancer Centre, Garbary 15, 61-866 Poznan, Poland; igor.piotrowski@wco.pl (I.P.); julia.ostapowicz@wco.pl (J.O.); ola.jazikowska1@gmail.com (A.J.); wiktoria.suchorska@wco.pl (W.M.S.); 3 Doctoral School, Poznan University of Medical Sciences, Bukowska 70, 60-812 Poznan, Poland; niewinski.md@gmail.com; 4 Department of Head and Neck Surgery, Poznan University of Medical Sciences, Garbary 15, 61-866 Poznan, Poland; joanna.marchlewska@wco.pl (J.M.); michal.masternak@ucf.edu (M.M.M.); wgolus@ump.edu.pl (W.G.); 5 Department of Cancer Pathology, Greater Poland Cancer Centre, Garbary 15, 61-866 Poznan, Poland; agata.kubicka@wco.pl; 6 Department of Tumor Pathology and Prophylaxis, Poznan University of Medical Sciences, Garbary 15, 61-866 Poznan, Poland; 7 Department of Electroradiology, Poznan University of Medical Sciences, Garbary 15, 61-866 Poznan, Poland; 8 Institute of Human Biology and Evolution, Adam Mickiewicz University in Poznan, Uniwersytetu Poznanskiego 6, 61-614 Poznan, Poland; karolinacz22001@gmail.com; 9 Department of Laboratory Diagnostics, Greater Poland Cancer Centre, Garbary 15, 61-866 Poznan, Poland; danuta.procyk@wco.pl (D.P.); ewa.leporowska@wco.pl (E.L.); 10 Columbia Center for Translational Immunology and Burch-Lodge Center for Human Longevity, Department of Medicine, Columbia University Medical Center, 630 West 168th Street, New York, NY 10032, USA; mjy2118@cumc.columbia.edu; 11 Burnett School of Biomedical Sciences, College of Medicine, University of Central Florida, 6900 Lake Nona Blvd, Orlando, FL 32827, USA

**Keywords:** head and neck squamous cell carcinoma, cellular senescence, senescence-associated secretory phenotype, circulating lymphocytes, prognostic biomarkers

## Abstract

Senescence-associated secretory phenotype (SASP) signaling, along with key markers such as *P16^INK4a^/CDKN2A* and *LMNB1*, has not been systematically studied in circulating lymphocyte subsets in head and neck squamous cell carcinoma (HNSCC). This study aimed to evaluate SASP-related genes and senescence markers in peripheral CD4^+^, CD8^+^, and CD19^+^ cells and assess their clinical relevance. Expression of *IL-6*, *IL-1β*, *TNFα*, *CXCL1*, *P16^INK4a^*/*CDKN2A*, and *LMNB1* was measured by RT-qPCR in sorted lymphocytes from 58 HNSCC patients at baseline, 31 post-treatment, and 13 controls. Statistical analyses included nonparametric tests, correlation analyses, and survival models (Kaplan–Meier, Cox regression). In the results, *LMNB1* was significantly upregulated in all lymphocyte subsets of HNSCC patients. *IL-6*, *CXCL1*, and *IL-1β* were elevated in CD4^+^ T cells. A coordinated co-expression network involving *IL-6*, *CXCL1*, *IL-1β*, *P16^INK4a^*/*CDKN2A*, and *LMNB1* was observed. Clinically, *IL-6* in CD8^+^ T cells was associated with higher nodal stage and worse survival, while *CXCL1* in CD19^+^ B cells independently predicted survival. No differences were found between pre- and post-treatment samples. Circulating lymphocytes in HNSCC display coordinated expression of selected senescence-associated genes, with *IL-6* and *CXCL1* as candidate prognostic biomarkers linked to tumor progression that warrant further validation.

## 1. Introduction

Head and neck squamous cell carcinoma (HNSCC) is an immunosuppressive malignancy in which peripheral blood immune profiling has attracted increasing attention as a minimally invasive tool for patient stratification [1,2]. While the tumor immune microenvironment has been extensively characterized, the transcriptional landscape of defined circulating lymphocyte subsets at diagnosis remains poorly understood [1,2]. Clinically, treatment of HNSCC is known to induce systemic immune changes. Both surgery and radiotherapy can alter blood cell counts, inflammatory markers, and lymphocyte composition, reflecting therapy-driven immune remodeling [3,4]. Clinical interventions may also contribute to accelerated biological aging, potentially through mechanisms such as increased cellular senescence, chronic inflammation, and immune exhaustion [4]. In particular, radiotherapy-associated lymphopenia and changes in the neutrophil-to-lymphocyte ratio (NLR) have been linked to clinical outcomes, underscoring the prognostic value of systemic inflammation [3,5,6]. Despite this, the relationship between systemic immune alterations and senescence-associated signaling in circulating immune cells remains insufficiently defined, particularly at the level of specific lymphocyte subsets in HNSCC.

Cellular senescence is a complex stress response characterized by durable cell-cycle arrest, triggered by diverse stimuli including genotoxic stress, oncogenic signaling, mitochondrial dysfunction, oxidative stress, and—particularly relevant in oncology—cytotoxic therapy and ionizing radiation [7,8]. Despite their proliferative arrest, senescent cells remain metabolically active and undergo extensive phenotypic remodeling, most notably through acquisition of the senescence-associated secretory phenotype (SASP), a pro-inflammatory program composed of cytokines, chemokines, growth factors, and proteases capable of reshaping tissue microenvironments [9,10]. Our previous study demonstrated that elevated expression of senescence-associated secretory phenotype (SASP) genes in HNSCC is associated with adverse clinicopathological features, including higher tumor stage and nodal involvement, suggesting a potential role of senescence-related signaling in tumor progression and prognosis [11]. However, the contribution of cellular senescence to systemic immune alterations in HNSCC remains largely unexplored.

The biological effects of senescence are highly context-dependent. In early carcinogenesis, senescence may act as a tumor-suppressive mechanism by limiting the expansion of damaged cells; however, the persistence of senescent cells can promote chronic inflammation, extracellular matrix remodeling, and tumor progression [12,13]. In HNSCC, therapy-induced senescence (TIS) has emerged as a relevant consequence of radiotherapy and systemic treatment. Radiation can induce senescence in both tumor and stromal compartments and activate SASP signaling, thereby contributing to microenvironmental remodeling characterized by persistent inflammatory signaling and altered immune cell recruitment, collectively fostering an immunosuppressive niche that supports tumor persistence and therapeutic escape [14,15]. In addition, growing evidence from aging research highlights the broader systemic consequences of senescence and its links to aging and disease susceptibility [13].

Importantly, senescence is closely linked with immune regulation. Senescent cells can be recognized and eliminated through immune-mediated mechanisms (“senescence surveillance”), with SASP factors facilitating recruitment and activation of immune effectors such as natural killer cells, macrophages, and T lymphocytes [16,17]. Conversely, chronic SASP signaling may drive immune dysregulation, sustaining inflammation and promoting immune evasion [18]. Notably, certain SASP components, including matrix metalloproteinases (MMPs), can cleave cell surface molecules involved in immune recognition, thereby impairing the efficient clearance of senescent cells. In addition, immune cells themselves can acquire senescent or senescence-like phenotypes during chronic antigen exposure and following oncologic therapy, leading to impaired proliferative capacity and altered effector function [19,20]. In HNSCC, highly differentiated T-cell subsets—such as CD28^−^ and/or CD57^+^ populations—have been explored as markers of immune remodeling [2].

Peripheral blood CD4^+^ T cells, CD8^+^ T cells, and CD19^+^ B cells play distinct roles in anti-tumor immunity and systemic inflammatory signaling. In this context, *IL-6*, *IL-1β*, *TNFα*, and *CXCL1* were selected as representative components of SASP, reflecting the pro-inflammatory signaling associated with senescent cells rather than senescence itself, while their expression may indicate tumor-induced systemic immune reprogramming [21,22]. In contrast, *P16^INK4a^*/*CDKN2A* and *LMNB1* were included as established markers of the senescent state, which are increasingly recognized as relevant to cancer-associated immune dysfunction [23,24]. Together, these genes enable the assessment of both cellular senescence and its associated inflammatory response [9,25]. However, their expression in circulating immune cell subsets of HNSCC patients has not been systematically characterized.

In this study, we analyzed the expression of these six genes in sorted CD4^+^ T cells, CD8^+^ T cells, and CD19^+^ B cells from HNSCC patients at baseline (T0) and after therapy (T1), as well as from healthy controls (CTR). These data were evaluated in the context of clinical parameters and longitudinal overall survival to identify expression patterns associated with disease status and patient outcomes. Notably, *P16^INK4a^* expression in peripheral blood mononuclear cells (PBMCs) and immune subsets has been widely used as a surrogate biomarker of biological aging, with studies showing its age-associated increase, particularly in T-cell compartments (e.g., CD3^+^ cells), supporting its relevance as a marker of systemic senescence [23].

## 2. Materials and Methods

### 2.1. Patient Samples

Whole blood samples were collected from head and neck squamous cell carcinoma patients who were qualified for tumor surgical resection in the Department of Head and Neck Surgery, Poznan University of Medical Sciences, The Greater Poland Cancer Centre. The study was approved by the Local Ethical Committee of Poznan University of Medical Sciences (no. 521/21). The exclusion criteria comprised the presence of a second primary malignancy, distant metastasis, and any history of prior oncological treatment, including radiotherapy or chemotherapy. The characterization of the total study cohort is presented in Table 1.

### 2.2. Study Design

Of the 63 enrolled patients, 58 were included in the final analyses; four were excluded due to insufficient biological material or a change in the planned treatment protocol. Of the 58 patients, 58 T0 samples (baseline; before surgery) and 31 T1 samples (post-treatment) of sufficient quality were obtained. Post-treatment samples were collected between 2 and 4 weeks after completion of adjuvant therapy. Peripheral blood was collected into EDTA tubes and peripheral blood mononuclear cells (PBMCs) were isolated by density gradient centrifugation using Ficoll-Paque™ PLUS (Cytiva, Uppsala, Sweden). CD4^+^ T cells, CD8^+^ T cells, and CD19^+^ B cells were subsequently purified by fluorescence-activated cell sorting (FACS). Gene expression of six targets—*IL-6*, *IL-1β*, *TNFα*, *CXCL1*, *P16^INK4a^*/*CDKN2A*, and *LMNB1*—was quantified by RT-qPCR in each sorted fraction. Expression data were analyzed in relation to clinical and hematological parameters including tumor stage (T, N), histological grade (G), anatomical site, adjuvant treatment, peripheral blood counts, and overall survival.

### 2.3. Cell Preparation/PBMCs Isolation

Peripheral blood was collected into S-Monovette^®^ EDTA K3E/9 mL tubes (Sarstedt AG & Co. KG, Nümbrecht, Germany). Blood was diluted 1:1 with PBS and carefully layered onto 10 mL Ficoll-Paque Ficoll-Paque™ PLUS (Cytiva, Uppsala, Sweden) in 50 mL tubes. Samples were centrifuged at 400× *g* for 30 min (RT, low brake). The PBMC layer was collected immediately, resuspended in PBS, and washed (300× *g*, 10 min, RT). For storage, PBMCs were cryopreserved in FBS with 10% DMSO at −80 °C.

### 2.4. Flow Cytometry and Cell Sorting

Prior to staining, PBMCs were resuspended in FACS buffer (Ca^2+^/Mg^2+^-free PBS supplemented with 2% FBS and 2 mM EDTA). Surface staining was performed using the following antibodies: anti-CD4-PE (clone 13B8.2, cat. no. A07751; Beckman Coulter, Marseille, France), anti-CD8-APC (clone B9.11, cat. no. IM2469; Beckman Coulter, Marseille, France), and anti-CD19-PerCp (clone LT19, cat. no. PC-305-T100; EXBIO Praha, a.s., Vestec, Czech Republic) and incubated for 20 min at 4 °C in the dark. Following staining, cells were washed twice and resuspended in Ca^2+^/Mg^2+^-free PBS prior to sorting.

Fluorescence-activated cell sorting was performed on a BD FACSAria™ I cell sorter using BD FACSDiva™ software v6.1.3 (both BD Biosciences, San Jose, CA, USA). Cells were gated on FSC/SSC scatter properties to exclude debris and presumed non-viable cells; a dedicated viability dye was not used. CD4^+^, CD8^+^, and CD19^+^ populations were then sorted as discrete fractions into RNase-free collection tubes. Sorted cells were centrifuged immediately and stored at −80 °C until RNA extraction.

### 2.5. RNA Isolation, Reverse Transcription, and Real-Time Quantitative Polymerase Chain Reaction (rt-qpcr) Analysis

Total RNA was isolated using the RNeasy Mini Kit (Qiagen, Hilden, Germany) according to the manufacturer’s protocol. The cDNA was synthesized with RevertAid First Strand cDNA Synthesis Kit (Thermo Fisher Scientific Baltics, Vilnius, Lithuania) using 100 ng of total RNA, oligo dT primers, and random hexamer primers. The real-time quantitative polymerase chain reaction for *IL6*, *IL-1β*, *CXCL1*, *TNFα*, *LMNB1,* and *P16^INK4a^* gene expression analysis was conducted with a PowerTrack SYBR Green Master Mix (Thermo Fisher Scientific, Vilnius, Lithuania) using the CFX96 Real-Time System (Bio-Rad Laboratories, Hercules, CA, USA). The reaction conditions for all amplicons were as follows: initially 95 °C for 15 min, followed by 40 cycles at 95 °C for 10 s, 60–62 °C (depending on the primers used) for 10 s, and 72 °C for 10 s. Gene expression was normalized to the GAPDH reference gene, and relative expression levels were determined using the Pfaffl method. The primer sequences used in this study are listed in Supplementary Table S1.

### 2.6. Statistical Analysis

Statistical analysis was performed using GraphPad Prism 10.4.1 software, and results with *p* < 0.05 were considered statistically significant. For the RT-qPCR analysis, we obtained gene expression data of six genes in 309 samples. The samples in which no reference gene expression was detected were excluded from analysis. Among the samples with reference gene expression, there were 72% non-detects. We observed that the average ΔCt for each gene/cell type combination correlated strongly with a higher fraction of non-detects (Spearman correlation *p* ≤ 0.0007, r = 0.7239, Supplementary Figure S1), which suggested that non-detects were not missing at random, and omitting those values would introduce significant bias. In order to reduce this bias, we decided to take a two-way approach to analysis: first, we analyzed the expression data after dichotomization (samples were either positive or negative for expression of a specific gene), and secondly, as continuous data. In order to analyze gene expression as continuous data, including non-detects, we used an imputation approach. For the measurements where the gene of interest was not detected, the Ct was replaced with the cycle equal to the number of cycles for which the reaction was run. Gene expression was then calculated using the Pfaffl method. The data were log-transformed before statistical analyses, as the log-transformed data followed a normal distribution more closely. The normality of the data distribution was assessed using the Shapiro–Wilk test. For the comparison between two groups, we used Student’s *t*-test (if data followed a normal distribution) or the Mann–Whitney U test (if data did not follow a normal distribution). We used Fisher’s exact test to compare the distribution of samples with and without gene expression between control and HNSCC groups. To determine the correlation between genes, we calculated Pearson correlation coefficients (data following normal distribution) or nonparametric Spearman correlation (data not following normal distribution). To analyze the effect of senescence marker expression on clinical and hematological parameters, we used Student’s *t*-test or Mann–Whitney U test to compare two groups—one with senescence marker expression and one without the senescence marker expression. The effect of senescence marker expression on survival was analyzed in two ways. Kaplan–Meier survival analysis was conducted to compare the survival of HNSCC patients with and without the marker expression. We used Cox proportional hazard regression analysis to assess how gene expression impacted survival.

## 3. Results

### 3.1. LMNB1, IL-6, IL-1β Are Significantly Upregulated in CD4^+^ Circulating Lymphocytes of HNSCC Patients Compared to Healthy Controls

To determine whether senescence-associated gene expression differed between HNSCC patients and healthy individuals, we compared relative expression values of six target genes (*IL-6*, *IL-1β*, *TNFα*, *CXCL1*, *P16^INK4a^*/*CDKN2A*, and *LMNB1*) across three lymphocytes subsets (CD4^+^, CD8^+^, CD19^+^) at baseline (T0, *n* = 58), after adjuvant treatment (T1, *n* = 31) and healthy controls (CTR, *n* = 13). First, we assessed whether the proportion of patients with detectable gene expression differed between groups; patients were classified as expression-positive or expression-negative for each gene, and proportions were compared between HNSCC (T0) and CTR, and between HNSCC (T0) and HNSCC (T1) groups using Fisher’s exact test. In CD4^+^ T cells, the HNSCC group had a significantly higher fraction of samples with *LMNB1* expression compared to controls (*p* = 0.0061). A similar abundance of *LMNB1* positivity in HNSCC samples was observed in CD8^+^ T cells (*p* = 0.0280). For CD19^+^ T cells, we observed a similar trend regarding *LMNB1*; however, the difference was not statistically significant (*p* = 0.0572). Expression of *IL-1β* was detected in more control samples of CD4^+^ T cells and CD19^+^ T cells compared to HNSCC samples; however, the difference was not statistically significant (*p* = 0.0549 and *p* = 0.0504, respectively). When HNSCC samples before and after therapy were compared, we observed a significantly lower fraction of CD4^+^ T cell samples positive for *IL-1β* expression post-therapy (*p* = 0.0391). The results of SASP expression analyses conducted on dichotomized data are presented in Supplementary Table S2.

**Figure 1:**
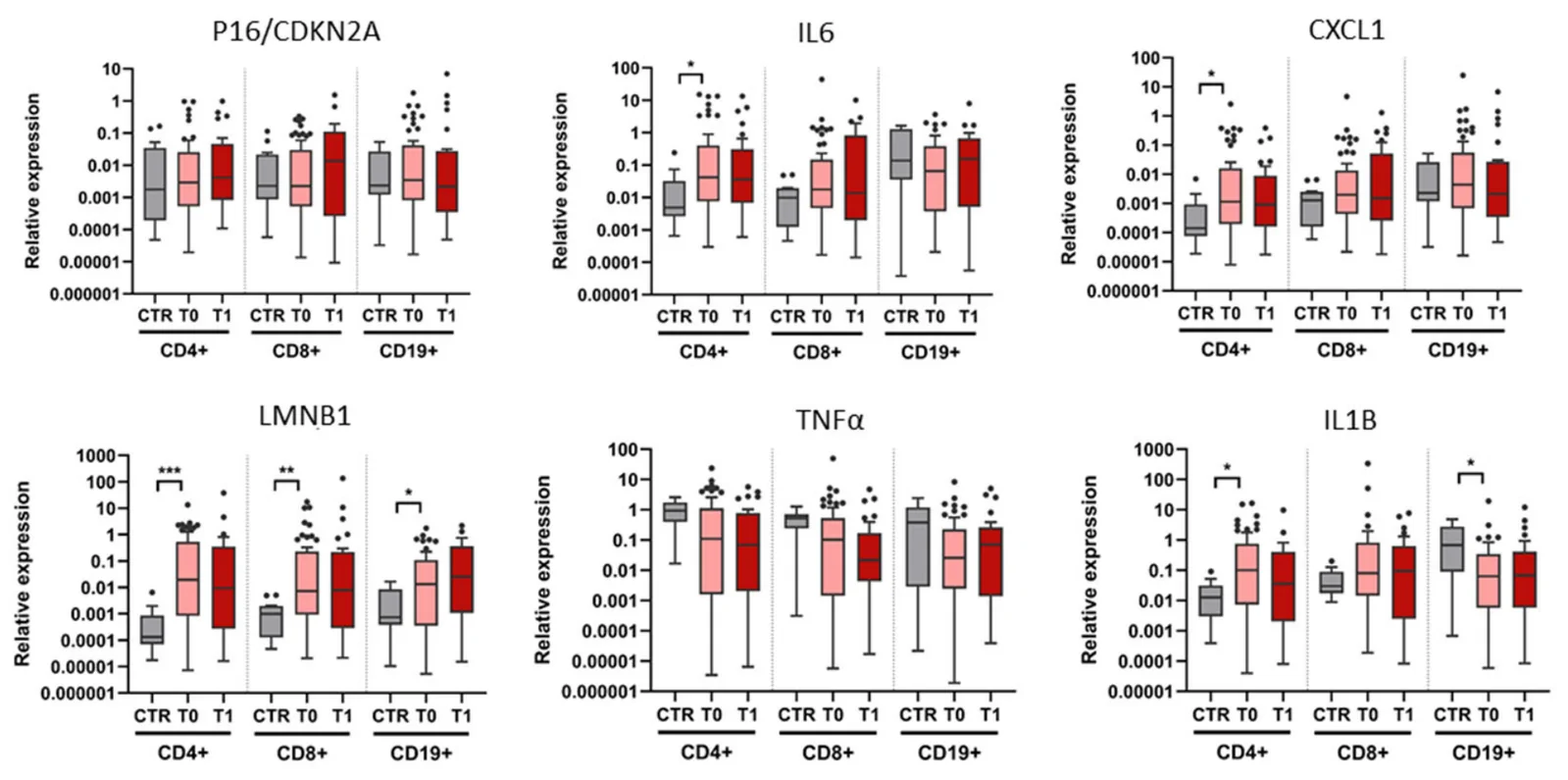
Senescence-associated and inflammatory gene expression in circulating CD4^+^, CD8^+^, and CD19^+^ lymphocyte subsets of HNSCC patients and healthy controls. The relative expression levels of *P16^INK4a^*/*CDKN2A*, *LMNB1*, *IL-1β*, *IL-6*, *CXCL1*, and *TNFα* in FACS-sorted CD4^+^ T cells, CD8^+^ T cells, and CD19^+^ B cells from healthy controls (CTR, *n* = 13), HNSCC patients before treatment (T0, *n* = 58), and HNSCC patients after treatment (T1, *n* = 31). Individual data points are overlaid on each box; each dot represents a separate sample from an individual participant. Boxes represent the interquartile range; horizontal lines within boxes indicate medians. Gene expression was normalized to *GAPDH* using the Pfaffl method. Statistical comparisons between CTR and T0 were performed using the Mann–Whitney U test or unpaired *t*-test as appropriate; significance is indicated as * *p* < 0.05, ** *p* < 0.01, *** *p* < 0.001. CTR, healthy controls; T0, pre-treatment; T1, post-treatment.

When expression data were analyzed as continuous, we observed that *LMNB1* expression was significantly elevated in HNSCC patients compared to controls in CD4^+^ T cells (*p* = 0.0003), CD8^+^ T cells (*p* = 0.0087), and CD19^+^ B cells (*p* = 0.046). *IL-6* and *IL-1β* expression was significantly higher in HNSCC patients in CD4^+^ cells (*p* = 0.0229, *p* = 0.0221, respectively). Additionally, lower expression of *IL-1β* was observed in the CD19^+^ B cell subset (*p* = 0.0267). We also observed a significantly higher expression of *CXCL1* in CD4^+^ T cells (*p* = 0.0346); however, the data had a high fraction of non-detects. The analysis on dichotomized data did not confirm this difference. No statistically significant differences in relative expression levels were observed for *P16^INK4a^* and *TNFα* across any cell subset (Figure 1). Also, no statistically significant differences in gene expression were observed between pre-therapy and post-therapy samples, across any gene and cell subset tested. Complete expression data are available in Supplementary Table S3.

### 3.2. Co-Expression of Senescence-Associated Genes in Circulating Lymphocyte Subsets of HNSCC Patients

**Figure 2:**
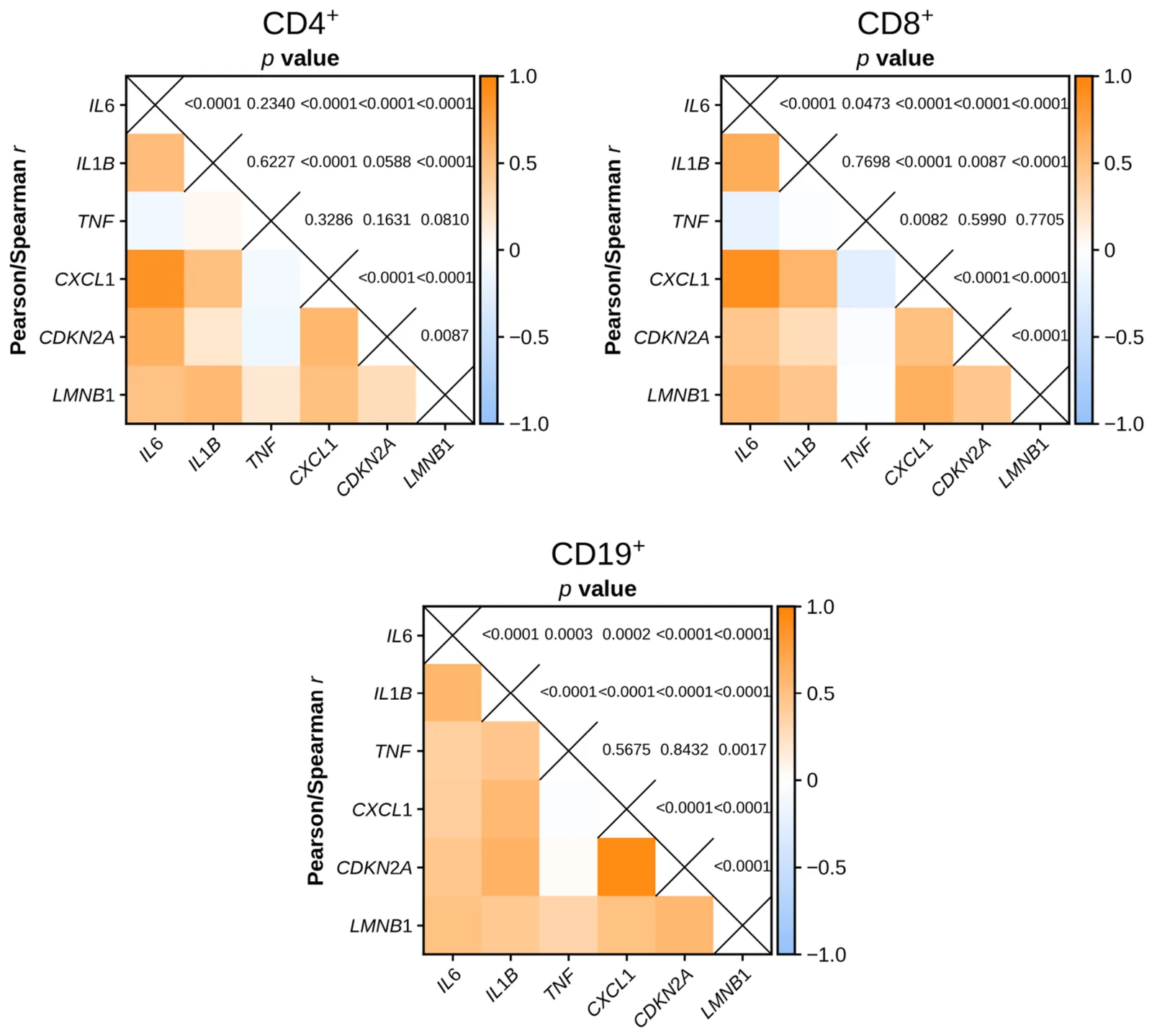
Pairwise gene expression correlations within CD4^+^, CD8^+^, and CD19^+^ lymphocyte subsets of HNSCC patients and healthy controls. Heatmaps displaying pairwise Pearson and Spearman correlation coefficients (r) between relative expression values of *IL-6*, *IL-1β*, *TNFα*, *CXCL1*, *P16^INK4a^*/*CDKN2A*, and *LMNB1* in FACS-sorted CD4^+^ T cells, CD8^+^ T cells, and CD19^+^ B cells from HNSCC patients at baseline (T0, *n* = 58). The upper triangle shows p-values; the lower triangle shows correlation coefficients (r) represented by color intensity on a diverging scale from −1 (blue, negative correlation) to +1 (orange, positive correlation). Diagonal cells are marked with a cross. Pearson or Spearman correlation was selected based on the normality of distribution.

To characterize the internal structure of gene co-expression within each lymphocyte subset, we performed pairwise Pearson and Spearman correlation analyses of expression values at T0. Results are presented as heatmaps in Figure 2. *IL-6* and *CXCL1* exhibited the strongest and most consistent co-expression across all three subsets (CD4^+^, CD8^+^, CD19^+^: all *p* < 0.0001). *IL-6* and *IL-1β* were significantly correlated in all subsets (CD4^+^, CD8^+^, CD19^+^: all *p* < 0.0001), as were *IL-1β* and *CXCL1* (CD4^+^, CD8^+^, CD19^+^: all *p* < 0.0001). *P16^INK4a^* and *LMNB1* were significantly correlated with each other in all three subsets (CD4^+^: *p* = 0.0087; CD8^+^: *p* < 0.0001; CD19^+^: *p* < 0.0001). *P16^INK4a^* and *LMNB1* each showed significant associations with both *IL-6* (CD4^+^: both *p* < 0.0001; CD8^+^: both *p* < 0.0001; CD19^+^: both *p* < 0.0001) and *CXCL1* (CD4^+^, CD8^+^ both *p* < 0.0001; CD19^+^: *p* = 0.0002), extending the co-expression cluster to encompass all five markers. The *IL-1β–P16^INK4a^* correlation was borderline in CD4^+^ cells (*p* = 0.0588) but reached significance in CD8^+^ (*p* = 0.0087) and CD19^+^ (*p* < 0.0001) subsets. In contrast, *TNFα* showed no significant correlation with *IL-6*, *IL-1β*, or *CXCL1* in CD4^+^ cells (*p* = 0.234, *p* = 0.623, *p* = 0.329, respectively), nor with *IL-1β*, *P16^INK4a^*, or *LMNB1* in CD8^+^ cells (*p* = 0.766, *p* = 0.599, *p* = 0.771), but a weak correlation with *IL-6* (*p* = 0.047). In CD19^+^ cells, *TNFα* showed no significant correlation with *CXCL1* (*p* = 0.568) or *P16^INK4a^* (*p* = 0.843), and a weak but significant association with *LMNB1* (*p* = 0.0017). Taken together, *TNFα* is consistently dissociated from the *IL-6*/*CXCL1*/*IL-1β*/*P16^INK4a^*/*LMNB1* co-expression cluster across all subsets, suggesting it is regulated through an independent program. Complete correlation data are available in Supplementary Table S4.

### 3.3. Gene Expression Status Is Associated with Clinical and Hematological Parameters in a Specific Cells’ Subset

To assess the clinical relevance of gene expression status, patients at baseline (T0) were stratified into expression-positive and expression-negative groups for each gene, and clinical as well as hematological parameters were compared within individual immune cell subsets (Table 2).

In CD4^+^ T cells, *IL-6* positivity was associated with lower monocyte counts (*p* = 0.0378). In contrast, *TNF-α* and *P16^INK4a^* negativity were associated with higher monocyte counts (*p* = 0.0346 and *p* = 0.0293, respectively), while *P16^INK4a^*-negative patients also showed higher basophil counts (*p* = 0.0239). Additionally, *IL-1β*-negative patients were approximately 6 years older than *IL-1β*-positive individuals (*p* = 0.0163).

In CD8^+^ T cells, *IL-6* positivity was associated with higher nodal stage (*p* = 0.0058), and *LMNB1* positivity with higher tumor stage (*p* = 0.0093). *IL-1β*-negative patients were older (by approximately 6 years; *p* = 0.0161), whereas *P16^INK4a^*-negative patients were younger (by approximately 7 years; *p* = 0.0364). Moreover, *P16^INK4a^* negativity was associated with higher monocyte counts (*p* = 0.0052).

In CD19^+^ B cells, *IL-6* positivity was associated with higher lymphocyte (*p* = 0.0116) and basophil counts (*p* = 0.0326). Complete data on associations between gene expression and clinical and hematological parameters are available in Supplementary Table S5.

No significant differences in the fraction of gene-positive samples were observed when comparing different primary tumor localizations (anatomical site within the head and neck region) across expression-positive vs. expression-negative groups for any gene in any subset (Supplementary Table S6).

### 3.4. IL-6 Expression in CD8^+^ Cells and CXCL1 Expression in CD19^+^ Cells Are Associated with Overall Survival

**Figure 3:**
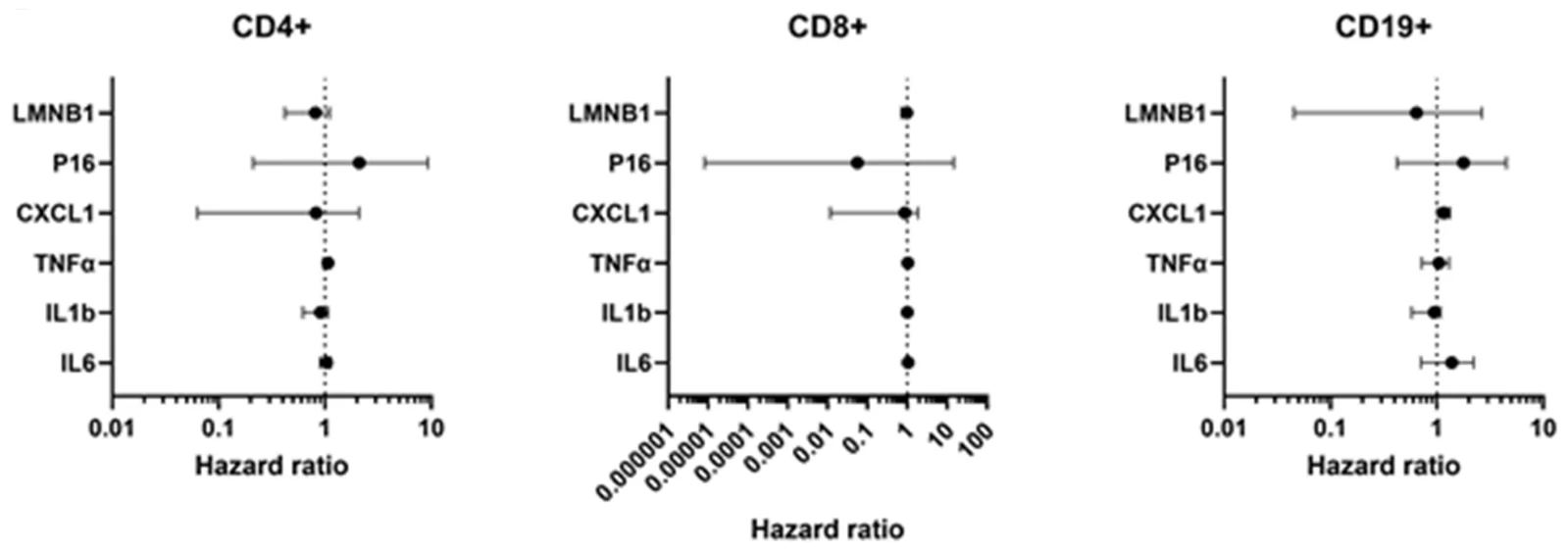
Association of *IL6*, *IL-1β*, *TNFα*, *CXCL1*, *P16^INK4a^*, and *LMNB1* gene expression status with overall survival. Forest plots showing hazard ratios (HR) with 95% confidence intervals from univariate Cox proportional hazards models for all analyzed gene–subset combinations in CD4^+^ T cells, CD8^+^ T cells, and CD19^+^ B cells. Most variables were not significantly associated with survival, as indicated by confidence intervals crossing 1. Notably, *IL-6* expression in CD8^+^ T cells and *CXCL1* expression in CD19^+^ B cells showed significant associations with overall survival.

To assess the prognostic relevance of gene expression patterns, Kaplan–Meier survival analysis with log-rank testing and univariate Cox proportional hazards models were performed for all gene–subset combinations at T0. The Kaplan–Meier survival analysis did not show any significant differences between survival of patients with vs. without SASP gene expression for any of the cell types (Supplementary Table S7). Among all analyzed variables, *IL-6* expression in CD8^+^ T cells was significantly associated with overall survival in univariate Cox regression (*p* = 0.0313), although the effect size was moderate. Across all subsets, most gene expression variables were not significantly associated with survival, as indicated by hazard ratios close to 1 and wide confidence intervals. Notably, *CXCL1* expression in CD19^+^ B cells was also significant in univariate Cox analysis (*p* = 0.0055), with lower expression associated with improved survival (Figure 3). However, this finding should be interpreted with caution due to a substantial proportion of missing data for those two genes, and no observed difference when dichotomized data were analyzed. Since the expression levels of *IL-6* and *CXCL1* were possibly linked with patient survival, we also investigated whether the combination of the two markers can be used for survival prediction. We performed COX regression analysis with *IL-6* expression in CD8^+^ T cells and *CXCL1* expression in CD19^+^ B cells as predictor variables. The model including both markers and their interaction showed similar robustness (*IL-6*: *p* = 0.0142, HR = 1.067 (0.9964–1.118); *CXCL1: p* = 0.0082, HR = 1.166 (1.022–1.330); interaction: *p* = 0.0592, HR = 6.786 (0.6393–39.95)). This model showed higher predictive value compared to the model including just *IL-6* (Akaike information criterion (AIC) lower by 4.423), and the model including just *CXCL1* (AIC lower by 2.283). The results point towards the high expression of these two markers as a predictor of worse clinical outcomes. Complete data on the effect of expression on overall survival are available in Supplementary Table S7.

## 4. Discussion

The present study provides a systematic characterization of senescence-associated and inflammatory gene expression in defined circulating lymphocyte subsets of HNSCC patients. Three principal findings emerge: (i) consistent upregulation of *LMNB1* across all lymphocyte populations, accompanied by increased *IL-1β* and *TNF-α* expression in T cells; (ii) the presence of a coherent co-expression module comprising *IL-6*, *CXCL1*, *IL-1β*, *P16^INK4a^*, and *LMNB1*, distinct from an independent *TNF-α*-associated program; and (iii) clinically relevant associations linking *IL-6* expression in CD8^+^ T cells and *CXCL1* expression in CD19^+^ B cells with overall survival. Together, these findings suggest that circulating lymphocytes in HNSCC patients exhibit a distinct senescence-associated transcriptional imprint, with components linked to tumor burden and clinical outcome.

### 4.1. LMNB1 as a Senescence Signature in Circulating T Cells

The most consistent finding across all three lymphocyte subsets was elevated *LMNB1* expression in HNSCC patients compared with healthy controls, accompanied by enrichment of *LMNB1*-positive individuals in the patient group. In classical cellular senescence, as defined in fibroblasts, epithelial cells, and other proliferating compartments, *LMNB1* is downregulated, and its loss is considered a canonical marker of senescent cell identity, contributing to nuclear lamina destabilization, chromatin redistribution, and cGAS–STING pathway activation [26,27]. However, the biology of LMNB1 in post-mitotic or slowly cycling immune cells differs fundamentally, and its regulation in lymphocytes has not been systematically characterized. Notably, LMNB1 can be upregulated in response to cellular stress, suggesting a stress-adaptive response in certain contexts [28]. At the same time, prolonged or sustained elevation of LMNB1 has been linked to adverse genomic consequences, including increased genomic instability [29]. Recent work has shown that *LMNB1* overexpression in cancer cells drives proliferation, chromatin remodeling, and therapy resistance [30], raising the possibility that elevated *LMNB1* in circulating T cells of HNSCC patients reflects not classical senescence but rather a stress-associated or oncology-driven remodeling of nuclear architecture. Also, the association between *LMNB1* positivity and higher tumor stage in CD8^+^ cells (*p* = 0.0093) is consistent with the hypothesis that *LMNB1* expression reflects the magnitude of systemic senescence-related signaling, which escalates with tumor burden [11,31].

### 4.2. SASP-Related Co-Expression Cluster

The strong co-expression of *IL-6*, *CXCL1*, *IL-1β*, *P16^INK4a^*, and *LMNB1* across all three lymphocyte subsets identifies a coherent transcriptional program consistent with a SASP-related state in circulating immune cells. The SASP encompasses a broad spectrum of downstream secreted factors co-regulated by NF-κB, C/EBPβ, and mTOR signaling [25,32]. The *IL-6*/*CXCL1* co-expression is among the strongest pairwise gene–gene correlations reported in human peripheral blood lymphocyte datasets. *IL-6* is a well-established SASP effector and a central mediator of the autocrine senescence reinforcement loop, sustaining senescent cell identity through JAK–STAT3 signaling [31]. CXCL1, a CXC-family chemokine, is co-regulated with *IL-6* in senescent cells and drives myeloid cell recruitment, a function that may contribute to the monocyte count associations observed in our dataset [32]. These findings extend our previous demonstration that SASP gene expression in HNSCC tumor tissue is associated with adverse clinicopathological features [11] by showing that a functionally related expression program is detectable in the circulating lymphocyte compartment. However, it should also be considered that latent viral infections may contribute to the observed senescence-associated profile. Chronic CMV infection, in particular, promotes the accumulation of terminally differentiated, senescent-like CD8^+^ T cells with a pro-inflammatory secretory phenotype, and could thus reinforce the coordinated SASP-like expression cluster identified here. As HPV and CMV status were not available, a contribution of these infections cannot be excluded and warrants assessment in future studies.

The consistent dissociation of *TNFα* from the *IL-6*/*CXCL1*/*IL-1β*/*P16^INK4a^*/*LMNB1* cluster is a reproducible finding in our dataset. TNFα is a pleiotropic cytokine with both pro- and anti-tumor functions, signaling through TNFR1 and TNFR2, which activate different intracellular pathways [33]. In contrast to *IL-6* and *IL-1β*, whose expression is mainly driven by NF-κB and supported by the cGAS–STING pathway, *TNFα* in lymphocytes can also be rapidly induced via antigen receptor signaling, independently of the senescence program [11].

The weak or negative correlations between *TNFα* and *IL-6*, *CXCL1*, and *IL-1β* across all subsets suggest that they are not part of a single inflammatory response. Instead, this supports the presence of at least two distinct inflammatory programs in circulating lymphocytes of HNSCC patients. The higher proportion of *TNFα*-positive individuals in healthy controls compared with HNSCC patients (CD4^+^, *p* = 0.0284) is consistent with previously described tumor-associated impairment of T-cell effector function in HNSCC [1,2].

### 4.3. IL-6 in CD8^+^ T Cells as a Prognostic Marker

The association between *IL-6* expression in CD8^+^ T cells, higher nodal stage (*p* = 0.0058), and shorter overall survival (Cox *p* = 0.0313) identifies this marker as a potential prognostic biomarker and links systemic senescence-associated signaling with tumor behavior. Elevated circulating *IL-6* has previously been associated with poor outcomes in HNSCC and other solid tumors [34,35], although most studies have focused on serum or plasma protein levels. In contrast, our findings suggest that *IL-6* gene expression in CD8^+^ T cells itself carries prognostic information, a finding not previously reported. This observation is biologically plausible, as CD8^+^ T cells with senescence-like or exhaustion-associated features and SASP-related gene expression may have reduced cytotoxic function while simultaneously contributing to a pro-inflammatory environment through *IL-6* signaling. This could support tumor progression and metastatic spread [2,19]. The additional association of *LMNB1* positivity with higher T stage in the same CD8^+^ subset further supports the idea of a functionally altered, senescence-associated CD8^+^ T-cell population with clinical relevance. These findings align with the emerging concept of immunosenescence in cancer [20,36]. More broadly, the role of the senescence-associated secretory phenotype in head and neck cancer has been described as context-dependent, acting as both a promoter and regulator of tumor progression [37,38].

### 4.4. CXCL1 Expression in B Cells

*CXCL1* expression in CD19^+^ B cells was associated with survival in Cox analysis. Given the known role of *CXCL1* in recruitment of immunosuppressive myeloid populations, this finding raises the possibility that a subset of B cells contributes to tumor-promoting inflammation [32]. Similarly to *IL-6* expression in CD8^+^ cells, *CXCL1* has not been directly linked to B cells, although in our previous research its expression was significantly increased in tumor tissue compared to the healthy margin [11]. However, this result should be interpreted with particular caution: as noted in the Results, *CXCL1* measurements in CD19^+^ B cells contained a high proportion of missing values, which limits the robustness of this association. Validation in a larger cohort will be required to confirm this observation.

### 4.5. Age-Related Associations and Immunosenescence

The consistent finding that *IL-1β*-negative patients in both CD4^+^ and CD8^+^ subsets were approximately six years older than *IL-1β*-positive patients is unexpected within the framework of classical inflammaging, the age-associated increase in systemic inflammatory tone typically characterized by elevated *IL-1β*, *IL-6*, and *TNFα* [11]. One possible explanation is that, in older HNSCC patients, the circulating lymphocyte has undergone more profound remodeling towards senescent or exhausted phenotypes, resulting in reduced *IL-1β* transcriptional capacity. Alternatively, older patients may have a higher proportion of *IL-1β*-unresponsive or terminally differentiated lymphocytes, consistent with the described reduction in NF-κB-responsive T-cell subpopulations in advanced immunosenescence [20]. In CD8^+^ cells, we observed the opposite relationship with age: *P16^INK4a^*-positive patients were older, which is consistent with *P16^INK4a^*/*CDKN2A* as a classical cell-cycle arrest marker that accumulates with replicative and oncogene-induced senescence [23]. Together, these age-associated patterns underscore the importance of including age as a covariate in future multivariate analyses.

### 4.6. Stability of Expression Across Therapy and Its Implications

When considering differences between gene expression pre-and post-treatment, only the fraction of *IL-1β*-positive CD4^+^ T cell samples was significantly lower after treatment. This could suggest a recovery into a less senescence-like phenotype after finishing treatment; however, this change was observed in only one of the senescence markers. This, in turn, may indicate that circulating lymphocyte profiles reflect relatively stable, patient-specific immune states rather than short-term treatment effects. Radiation-induced senescence in tumor and stromal compartments, which has been demonstrated in preclinical HNSCC models [14], may not be detectable as a transcriptional shift in circulating lymphocytes within the 2–4-week post-treatment window examined here. Also, only 31 of 58 patients provided paired T1 samples; therefore, statistical power for detecting modest therapy-induced changes is limited. Alternatively, therapy-induced changes may occur primarily in tissue compartments not captured in peripheral blood or may require longer follow-up to become detectable.

### 4.7. Limitations

There are several limitations of this study. First, the sample size was small and came from a single institution (58 patients at baseline), which reduces statistical power and means the results have not been validated in other cohorts. Related to this, the control and patient groups were not matched for age or sex; controls were younger and predominantly female, whereas patients were older and predominantly male. As both factors may modulate immune cell function, the case–control comparisons should be interpreted with caution and confirmed in larger, matched cohorts using age- and sex-adjusted models; importantly, the prognostic associations reported here were assessed within the patient group and are therefore not affected by this imbalance. Second, a significant proportion of non-detects among tested samples weakens the ability to reach conclusive results. To obtain more robust qPCR results, we conducted the analyses in two ways: on dichotomized expression data (detected expression and no detected expression) and on data calculated after Ct imputation with the maximum reaction cycle. Both approaches have their limitations and biases; however, when analysis results from both methods were consistent, we could conclude with higher certainty that the observed effects were not an artifact. In addition, *P16^INK4a^*/*CDKN2A* has two roles in HNSCC: it can reflect immune cell aging in lymphocytes, but it is also a marker associated with HPV-related tumors in cancer tissue [39]. Since HPV status was not available in this study, it is harder to fully interpret the *P16^INK4a^*/*CDKN2A* findings. Moreover, cytomegalovirus (CMV) serostatus was not assessed, although CMV infection is known to influence T-cell differentiation and senescence-associated features, including alterations in *P16^INK4a^*/*CDKN2A* expression. Also, there were no functional experiments to confirm that the identified lymphocytes are truly senescent or exhausted. Therefore, the term “senescence-associated” is based on gene expression patterns rather than direct functional evidence. Moreover, cellular senescence was assessed using a limited panel of two canonical markers (*P16^INK4a^*/*CDKN2A* and *LMNB1*) together with SASP-related cytokines (*IL-6*, *IL-1β*, *TNFα*, *CXCL1*), which reflect the pro-inflammatory SASP rather than the senescent state per se. A more comprehensive marker set—including, for example, SA-β-galactosidase activity, additional cell-cycle regulators, or DNA-damage markers—would be required to definitively establish a senescent phenotype, and the present results should therefore be interpreted as senescence-associated rather than as direct evidence of cellular senescence. Finally, bulk RT-qPCR was performed on broad CD4^+^, CD8^+^, and CD19^+^ populations, which yields an averaged signal and cannot resolve intra-population heterogeneity. Because subset-defining markers such as CD28^+^ and CD57^+^ were not included, the precise cellular source of the SASP-related signals cannot be identified and will require single-cell or marker-resolved approaches.

## 5. Conclusions

This study provides, to our knowledge, one of the first systematic characterizations of senescence-associated and inflammatory gene expression in FACS-sorted circulating CD4^+^ T cells, CD8^+^ T cells, and CD19^+^ B cells from HNSCC patients. *LMNB1* was consistently upregulated across all three lymphocyte subsets and was associated with higher tumor stage in CD8^+^ T cells. Importantly, this upregulation contrasts with the canonical loss of *LMNB1* in classical senescence, suggesting a non-canonical, stress-adaptive pattern that may reflect oncology-driven nuclear remodeling rather than a classical senescence marker. A coherent SASP-like co-expression cluster comprising *IL-6*, *CXCL1*, *IL-1β*, *P16*, and *LMNB1* was reproducibly identified across all lymphocyte populations, while *TNFα* was consistently dissociated from this cluster, raising the possibility of at least two distinct inflammatory programs in the circulating lymphocyte compartment of HNSCC patients. *IL-6* expression in CD8^+^ T cells at baseline was associated with higher nodal stage and shorter overall survival, suggesting a potential role as a prognostic biomarker at the intersection of immune senescence and tumor biology. *CXCL1* expression in CD19^+^ B cells showed a similar association, though this observation is preliminary and requires validation in larger cohorts. Gene expression profiles remained stable across the treatment period, suggesting they reflect individual baseline immune characteristics rather than dynamic therapy responses. This interpretation should be confirmed in larger, longitudinally powered studies.

These exploratory findings support the feasibility of senescence-associated profiling in HNSCC and identify *IL-6* in CD8^+^ cells and *CXCL1* in CD19^+^ cells as candidate prognostic biomarkers warranting prospective validation.

## Figures and Tables

**Table 1:** Clinical characteristics of the HNSCC cohort.

Characteristic	Total Number (*n*/%)
Total HNSCC patients	63
Male (M)	47 (74.6%)
Female (F)	16 (25.4%)
Age	
Mean age	64.1 years
Median age	64 years
Range	38–89 years
Anatomical site	
Oral cavity	43 (68.3%)
Larynx	20 (31.7%)
Tumor stage (T)	
T1	5 (7.9%)
T2	17 (27%)
T3	22 (34.9%)
T4	19 (30.2%)
Nodal stage (N)	
N0	28 (44.4%)
N1	8 (12.7%)
N2a	5 (7.9%)
N2b	4 (6.3%)
N2c	2 (3.2%)
N2 (unspecified)	4 (6.3%)
N3b	11 (17.5%)
N3 (unspecified)	1 (1.6%)
Histological grade (G)	
G1	14 (22.2%)
G2	35 (55.6%)
G3	14 (22.2%)
Adjuvant therapy	
None recorded	36 (60%)
Radiotherapy alone	17 (28.34%)
Radiochemotherapy	6 (10%)
Radioimmunotherapy	1 (1.66%)
Healthy controls	13
Age	
Mean age	52 years
Median age	53 years
Range	47–56 years
Sex	
Male (M)	3 (23.08%)
Female (F)	10 (76.92%)

**Table 2:** Associations between gene expression status and clinical and hematological parameters in HNSCC patients at baseline (T0).

Cell Population	Gene	Parameter	*p*-Value	Observation
CD4^+^	*IL-6*	Monocyte count	0.0378 *	Lower monocyte count in *IL-6*^−^ patients
	*IL-1β*	Age	0.0163 *	*IL-1β*^−^ patients ~6 years older
	*TNFα*	Monocyte count	0.0346 *	Higher monocyte count in *TNFα*^−^ patients
	*P16^INK4a^*	Monocyte count	0.0293 *	Higher monocyte count in *P16^−^* patients
	*P16^INK4a^*	Basophil count	0.0239 *	Higher basophil count in *P16^−^* patients
CD8^+^	*IL-6*	Nodal stage (N)	0.0058 **	Higher N stage in *IL-6^+^* patients
	*IL-6*	Neutrophil count	0.0253	Lower neutrophil count in *IL-6^−^* patients
	*IL-1β*	Age	0.0161 *	*IL-1β^−^* patients are ~6 years older
	*IL-1β* *P16^INK4a^*	Lymphocyte count Age	0.0195	Lower lymphocyte count in *IL-1β^−^* patients
			0.0364 *	*P16*^−^ patients are ~7 years younger
	*P16^INK4a^*	Monocyte count	0.0052 **	Higher monocyte count in *P16*^−^ patients
	*LMNB1*	Tumor stage (T)	0.0093 **	Higher T stage in *LMNB1*^+^ patients
CD19^+^	*IL-6*	Lymphocyte count	0.0116 *	Higher lymphocyte count in *IL-6*^+^ patients

* *p* < 0.05; ** *p* < 0.01. Mann–Whitney U test or unpaired t-test used as appropriate. Superscripts + and—denote expression-positive and expression-negative patient groups, respectively, as defined by detectable vs. undetectable gene expression.

## Data Availability

The data presented in this study are available on request from the corresponding author. The data are not publicly available due to privacy and ethical restrictions.

## Supplementary Materials

The following supporting information can be downloaded at: https://www.mdpi.com/article/10.3390/cells15161477/s1, Figure S1: Transcript abundance predicts non-detect frequency across gene/cell-type combinations. Table S1: Oligonucleotide sequences used in RT-qPCR analysis. Table S2: Comparison of SASP expression in circulating lymphocytes from healthy controls (CTR), head and neck squamous cell carcinoma patients before therapy (HNSCC T0) and after therapy (HNSCC T1). Table S3: Comparison of SASP expression in circulating lymphocytes from healthy controls (CTR), head and neck squamous cell carcinoma patients before therapy (HNSCC T0) and after therapy (HNSCC T1). Table S4: Correlation between the expression of SASP genes in circulating lymphocytes. Table S5: The effect of SASP gene expression (dichotomized as positive or negative) in circulating lymphocytes on clinical variables. Table S6: Comparison of SASP expression status in circulating lymphocytes from head and neck squamous cell carcinoma patients with oral and laryngeal cancer. Table S7: Survival prediction based on SASP gene expression levels in circulating lymphocytes.

## Abbreviations

The following abbreviations are used in this manuscript:

HNSCC	Head and neck squamous cell carcinoma
SASP	Senescence-associated secretory phenotype
RT-qPCR	Real-time quantitative polymerase chain reaction
FACS	Fluorescence-activated cell sorting
PBMCs	Peripheral blood mononuclear cells
NLR	Neutrophil-to-lymphocyte ratio
TIS	Therapy-induced senescence
CTR	Healthy controls
T0	Baseline (before treatment)
T1	Post-treatment
PBS	Phosphate-buffered saline
FBS	Fetal bovine serum
DMSO	Dimethyl sulfoxide
EDTA	Ethylenediaminetetraacetic acid
GAPDH	Glyceraldehyde-3-phosphate dehydrogenase
HPV	Human papillomavirus
HR	Hazard ratio
IL-6	Interleukin 6
IL-1β	Interleukin 1 beta
TNFα	Tumor necrosis factor alpha
CXCL1	C-X-C motif chemokine ligand 1
P16/CDKN2A	Cyclin-dependent kinase inhibitor 2A
LMNB1	Lamin B1
NF-κB	Nuclear factor kappa B
JAK	Janus kinase
STAT3	Signal transducer and activator of transcription 3
cGAS	Cyclic GMP-AMP synthase
STING	Stimulator of interferon genes
FSC	Forward scatter
SSC	Side scatter
CMV	Cytomegalovirus
