# Supplementary material for: Cellular Senescence-Associated Gene Expression in Circulating CD4+, CD8+, CD19+ Lymphocytes of HNSCC Patients: Associations with Clinical Parameters
Source: Cells. 2026 Aug 18;15(16):1477. doi: 10.3390/cells15161477 (PMC13511670; doi:10.3390/cells15161477)
Supplement: Supplementary file 1 [file cells-15-01477-s001.zip › supplementary figure KO.pdf]

Supplementary Figure S1

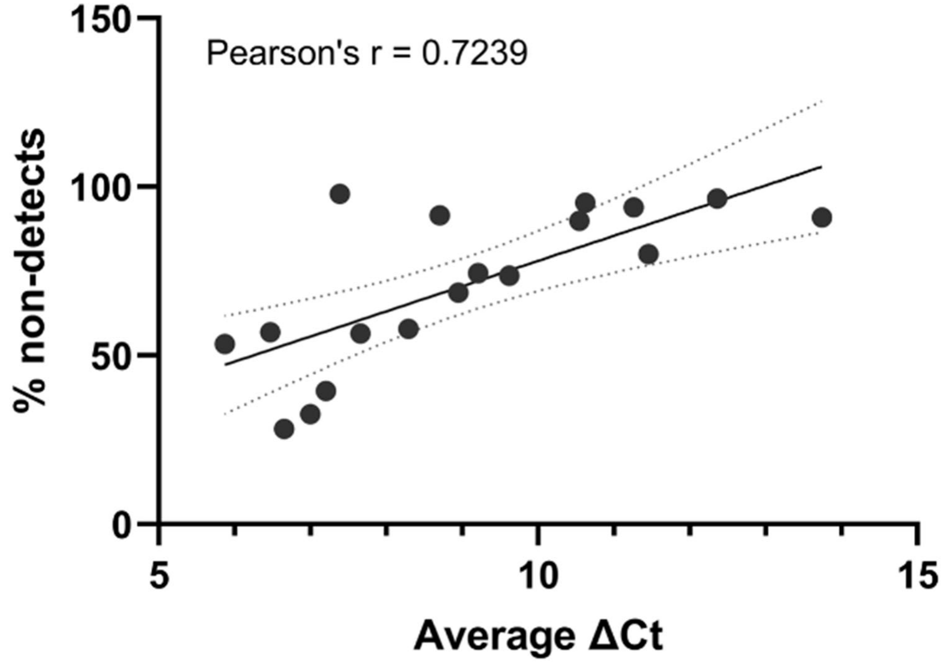

Supplementary Figure S1. Transcript abundance predicts non-detect frequency across gene/cell-type combinations. For each gene/cell-type combination, the mean ΔCt (lower values indicate higher expression) is plotted against the proportion of non-detects. Higher mean ΔCt values, corresponding to lower expression, were strongly associated with a greater fraction of non-detects (Spearman $r = 0.724$, $p < 0.0007$), indicating that non-detects occurred predominantly for low-abundance targets rather than reflecting random technical dropout.
